# A chemical screen underscores the essential role of STAT1-dependent IFNγ signaling to regulate HLA-I expression in cancer cells

**DOI:** 10.17912/micropub.biology.000697

**Published:** 2023-01-18

**Authors:** Myriam Barz, Bartlomiej Porebski, Pranauti Panshikar, Maria Häggbladd, Daniela Hühn, Oscar Fernandez-Capetillo

**Affiliations:** 1 Science for Life Laboratory, Division of Genome Biology, Department of Medical Biochemistry and Biophysics, Karolinska Institute, Stockholm, Sweden; 2 Genomic Instability Group, Spanish National Cancer Research Centre (CNIO), Madrid, Spain

## Abstract

The presentation of neoantigens by HLA-I is essential for the recognition of tumor cells by cytotoxic T cells. Transcriptionally, HLA-I expression is regulated by interferon-dependent activation of JAK/STAT signaling. Accordingly, mutations that inactivate this pathway are one of the main causes of resistance to cancer immunotherapies. Recent evidences indicate that HLA-I expression can be induced independently of IFN-signaling by the innate immune response. In this context, we performed an image-based screen to evaluate how more than 5,000 chemicals, including all medically available drugs plus many others in advanced preclinical development, influence HLA-I expression in STAT1-deficient cells. Our screening failed to identify any significant hits, suggesting that drug-dependent modulation of HLA-I expression is strictly dependent on IFN-signaling.

**Figure 1.**
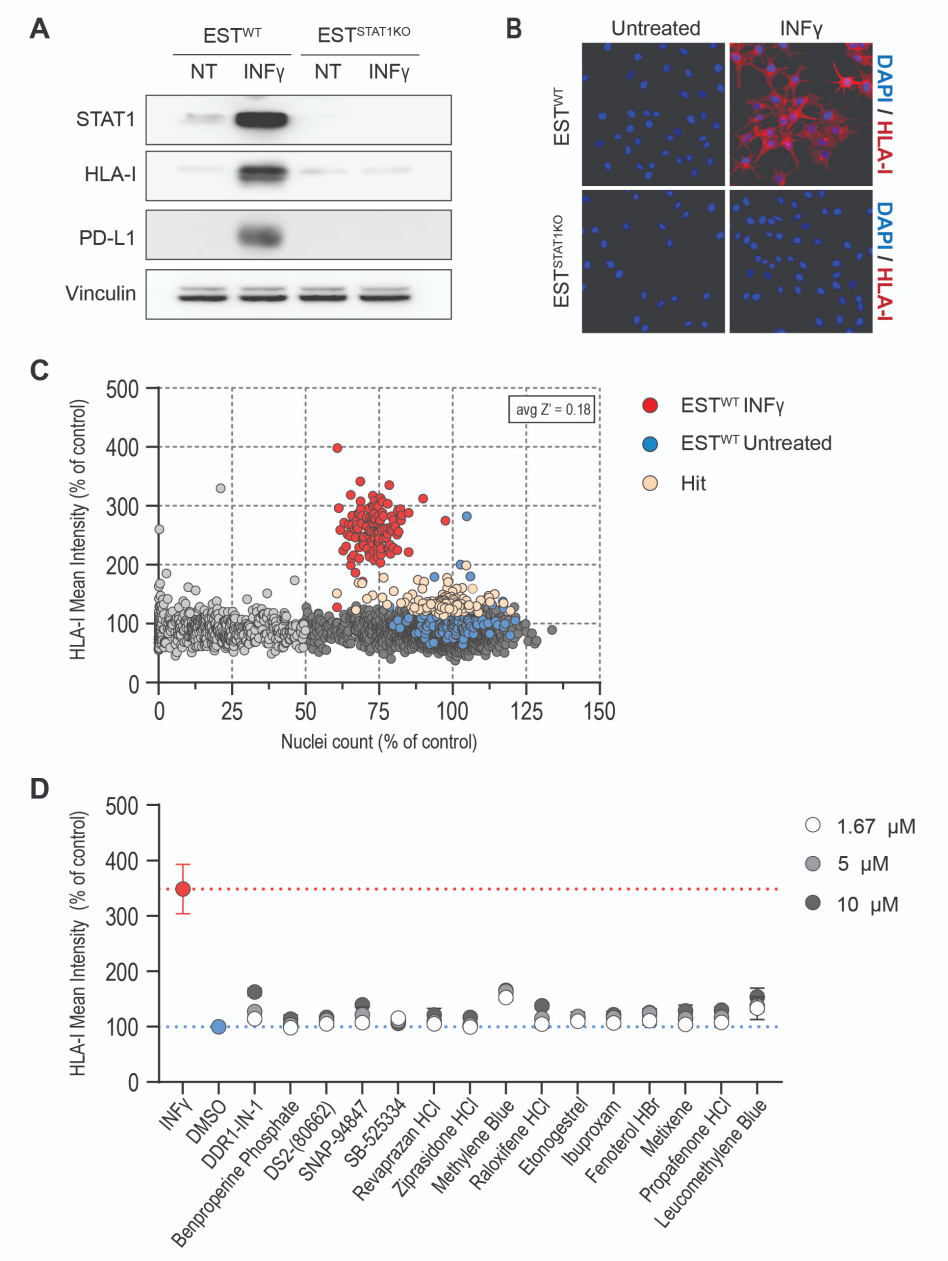
**Figure 1. A chemical screen for modulators of HLA-I expression.** (A) Western blot illustrating the absence of PD-L1 and HLA-I expression in STAT-1-deficient ESTDAB127 melanoma cells (EST ^STAT1KO^ ) in response to IFNγ (10 ng/mL, 72 hrs). (B) HLA-I expression (red), as measured by IF, in wild type and STAT-1-deficient cells in response to IFNγ (10 ng/mL, 72 hrs). Hoechst 33342 was used to stain DNA (blue). (C) Results of the primary screen of HLA-I regulators. EST ^STAT1KO^ melanoma cells were seeded in 384-well plates and treated with 5,278 drugs from the drug-repurposing hub collection at 5 μmM for 72 hrs. At this point, cells were fixed and processed for the detection of HLA-I by IF, using Hoechst 33342 to stain DNA. Wild type ESTDAB127 cells untreated (blue) or stimulated with IFNγ (red) were used as negative and positive controls, respectively, for HLA-I induction and detection. (D) HLA-I expression levels from 15 compounds from the secondary dose-response validation screen performed in EST ^STAT1KO^ cells. The secondary dose-response validation screen tested the effects of 115 compounds identified in the primary screen.

## Description

By disabling immune checkpoints, such as those regulated by cytotoxic T-lymphocyte-associated protein 4 (CTLA-4) or programmed cell death-1 (PD-1), cancer immunotherapies stimulate the activity of immune cells that can thereby promote the clearance of cancer cells (Waldman et al. 2020). This strategy has been one of the most promising developments in cancer therapy in recent decades, presenting impactful responses in patients with tumors of otherwise bad prognosis, such as melanoma. Unfortunately, and as it is the case with all therapies, resistance to immunotherapy limits its long-term efficacy and is an important medical problem (Kalbasi and Ribas 2020). Among the mechanisms of resistance to immune checkpoint inhibitors (ICIs), the most common is an impaired expression of the human leukocyte antigen class I (HLA-I), which is part of the major histocompatibility complex (MHC) that presents tumor neoantigens to cytotoxic CD8 ^+^ T cells (Gettinger et al. 2017). HLA-I expression is mainly regulated through type I and type II interferon-dependent activation of JAK/STAT-1 signaling (Hazini et al. 2021), and mutations that inactivate this signaling route have also been shown to disable the response to ICIs (Gao et al. 2016; Shin et al. 2017). Interestingly, recent works have revealed the existence of an IFNγ-independent mechanism that promotes HLA-I expression through activation of the innate immune system, although the relevance of this pathway to modulate HLA-I expression in cancer immunotherapy remains to be fully ascertained (Kalbasi et al. 2020; Such et al. 2020). Based on these observations, we wondered whether it is possible to trigger IFNγ-independent expression of HLA-I in cancer cells by using available drugs, which could potentially improve the efficacy of ICI therapies in tumors that have mutations that render them insensitive to IFNγ.

To address this, we decided to conduct a High-Throughput Microscopy (HTM)-based chemical screen, to evaluate the impact of more than 5,000 small molecules on HLA-I expression. This is the same strategy we recently used for evaluating how medically available drugs influence the expression of PD-L1, and which revealed that prolonged hormone therapy triggers the expression of PD-L1 and PD-L2 in estrogen receptor positive breast cancer cells (Huhn et al. 2022). As for the library, we used the one defined in the “Drug Repurposing Hub”, which is a curated open-access repository that includes all medically approved drugs plus many that have gone to clinical development yet failed to show efficacy in their primary objective (Corsello et al. 2017). To inactivate IFNγ-signaling, we used genome editing by CRISPR-Cas9 to generate STAT-1-deficient ESTDAB127 melanoma cells (EST ^STAT1KO^ , hereafter). The insensitivity to IFNγ of EST ^STAT1KO^ cells was confirmed by the absence of IFNγ-dependent induction of PD-L1 or HLA-I expression by western blotting ( **Fig. 1A** ). We then adapted the detection of HLA-I by immunofluorescence (IF) to high-throughput format in 384-well plates. Once again, IF confirmed the absence of IFNγ-dependent induction of PD-L1 or HLA-I expression in EST ^STAT1KO^ cells ( **Fig. 1B** ).

The screen was conducted by exposing cells to the compounds at 5 μM for 72 hrs, after which the plates were processed for the HTM-based quantification of HLA-I. Viability was also measured by quantifying nuclei numbers after staining DNA with Hoechst 33342. The results of the screen are illustrated in **Fig.1C** . As shown, it became evident that we could almost exclusively detect an induction of HLA-I expression in the controls (red dots), which were wild type ESTDAB127 cells stimulated with IFNγ. In contrast, very few compounds were able to do so in EST ^STAT1KO^ cells. After filtering-out toxic compounds (normalized viability <50%), we ran a dose-response validation screen with 115 compounds that had even a slight impact on HLA-I expression ( **Fig. 1C** , orange dots). However, this experiment failed to show a significant impact of any of the drugs tested on HLA-I expression, and the slight effects observed were mostly related to autofluorescence properties of the drugs (e.g. Methylene blue) ( **Fig. 1D** shows the validation screen results for the 15 compounds that had the biggest impact on HLA-I levels in the primary screen).

Restoring HLA-I expression would be a major advance to overcome the resistance to ICIs in cancer immunotherapy, and important efforts are currently being dedicated to this task. Unfortunately, and given that we used a large collection of drugs covering most biological pathways, our results suggest that the chemical-induction of HLA-I expression in cancer cells harboring mutations that disable IFNγ signaling is unlikely to succeed. To the very least, we can state that none of the available medicines can seemingly do so. Whether other strategies, such as gene therapies targeting the innate immune system, can achieve this important objective and overcome resistance to ICIs in cancer patients remains to be addressed.

## Methods

**Generation of STAT1-deficient cells**

The human uveal melanoma cell line ESTDAB127 from the European Collection of Authenticated Cell Cultures was used (ECACC 13012458). Cells were cultured in RPMI 1640 supplemented with 10% fetal bovine serum and 1% penicillin-streptomycin. STAT1-deficient cells (EST ^STAT1KO^ ) were generated by reverse transfection with a GFP-tagged Cas9 expressing plasmid carrying a sgRNA targeting human *STAT1* . GFP-positive cells were sorted by FACS followed by clonal expansion. STAT1-deficient clones were identified by western blotting using an anti-STAT1 antibody (#14994S, Cell Signaling Technology, 1:1000). For further validation of the absence of PD-L1 and HLA-I expression in STAT1 deficient cells treated with IFNγ (10 ng/mL, 72 hours) a western blot was carried out using anti-PD-L1 (#13684S, Cell Signaling Technology, 1:500) and anti-HLA-I (ab134189, Abcam, 1:1000) antibodies.

**Chemical screen**

Compounds of the “Drug Repurposing hub”, provided by the Chemical Biology Consortium Sweden (CBCS), were pre-spotted at 5 μM in 384-well plates (BD Falcon, 353962) using Echo®550 (Labcyte). The list of compounds is available in Extended Data 1. ESTDAB127 ^WT^ cells were seeded on top of the compounds at 500 cells per well, and EST ^STAT1KO^ at 750 cells per well. Plates were incubated at 37°C in 5% CO _2_ for 72 hours. Cells were fixed with 4% formaldehyde and washed with PBS. The Anti-HLA Class I antibody [W6/32] (ab23755, Abcam) (final 1:2000 in 3% BSA and 0.1% Tween) was added on top and incubated at 4°C overnight. Next day, cells were washed with PBS and incubated in a mixture of 3% BSA and 0.1% Tween containing 2 μM Hoechst 33342 (14433, Sigma-Aldrich), Phalloidin Alexa Fluor Plus 555 (A30106, Invitrogen) (1:20.000) and secondary anti-mouse Alexa647-conjugated antibody (A-21235, Thermo Fisher Scientific) (1:1000) for 1 hour at room temperature. Plate and liquid handling were performed using MultiFlo Dispenser (Integra), VIAFLO 384 (Integra) and HydroSpeed plate washer (Tecan).

Plates were imaged using an InCell Analyzer 2200 High Content Microscope with a 10x objective. Images were analysed using CellProfiler ( www.cellprofiler.org ). Nuclei were segmented using the Hoechst signal, cell shape was segmented using a Phalloidin signal and HLA-I intensity was measured in the entire cell. Analysis of high-content imaging data was performed using KNIME Analytics software ( www.knime.com ). Mean per-well intensity of HLA-I and mean per-well nuclei count were calculated using data from all 4 imaged fields and obtained values normalized within each plate to the mean value of DMSO-treated samples. Normalization was done separately for EST ^STAT1KO^ and EST ^WT^ samples. The Z-prime factor was calculated based on the HLA-I signal from DMSO-treated (negative control) and IFNγ-treated (positive control) EST ^WT^ cells separately for each plate. For hit calling, library-treated samples were filtered for toxicity (non-toxic if normalized viability > 50%) and then Z-scores were calculated based on normalized HLA-I intensity. Compounds with a Z-score above 2 were selected for secondary validation. The dose-response validation screen was done using the same protocol, in triplicates for each condition.

## Extended Data

Description: List of compounds from the library used in the screen. Resource Type: Collection. DOI: 10.22002/kn1c9-4g133
